# A New C-Xyloside Induces Modifications of GAG Expression, Structure and Functional Properties

**DOI:** 10.1371/journal.pone.0047933

**Published:** 2012-10-26

**Authors:** Emilie Vassal-Stermann, Albert Duranton, Annie F. Black, Gayane Azadiguian, Julien Demaude, Hugues Lortat-Jacob, Lionel Breton, Romain R. Vivès

**Affiliations:** 1 Institut de Biologie Structurale Jean-Pierre Ebel, Unité Mixte de Recherche (UMR) 5075, CNRS-CEA-Université Joseph Fourier, Grenoble, France; 2 L’Oréal Research and Innovation, Clichy, France; University of Patras, Greece

## Abstract

Proteoglycans (PGs) are critically involved in major cellular processes. Most PG activities are due to the large interactive properties of their glycosaminoglycan (GAG) polysaccharide chains, whose expression and fine structural features are tightly controlled by a complex and highly regulated biosynthesis machinery. Xylosides are known to bypass PG-associated GAG biosynthesis and prime the assembly of free polysaccharide chains. These are, therefore, attractive molecules to interfere with GAG expression and function. Recently, we have developed a new xyloside derivative, C-Xyloside, that shares classical GAG-inducing xyloside activities while exhibiting improved metabolic stability. We have previously shown that C-Xyloside had beneficial effects on skin homoeostasis/regeneration using a number of models, but its precise effects on GAG expression and fine structure remained to be addressed. In this study, we have therefore investigated this in details, using a reconstructed dermal tissue as model. Our results first confirmed that C-Xyloside strongly enhanced synthesis of GAG chains, but also induced significant changes in their structure. C-Xyloside primed GAGs were exclusively chondroitin/dermatan sulfate (CS/DS) that featured reduced chain size, increased O-sulfation, and changes in iduronate content and distribution. Surprisingly, C-Xyloside also affected PG-borne GAGs, the main difference being observed in CS/DS 4-O/6-O-sulfation ratio. Such changes were found to affect the biological properties of CS/DS, as revealed by the significant reduction in binding to Hepatocyte Growth Factor observed upon C-Xyloside treatment. Overall, this study provides new insights into the effect of C-Xyloside on GAG structure and activities, which opens up perspectives and applications of such compound in skin repair/regeneration. It also provides a new illustration about the use of xylosides as tools for modifying GAG fine structure/function relationships.

## Introduction

Proteoglycans (PGs) are glycoproteins abundantly found in the extracellular matrix (ECM) and at the cell surface, that are critically involved in a large array of cell functions, including cell adhesion, migration, proliferation and differentiation, embryo development, inflammation, pathogen infection or tumour growth and metastasis [1], [2], [3], [4]. These broad activities are mainly due to a strategic positioning at the interface between the cell and its surrounding environment, and to the ability of glycosaminoglycan (GAG) polysaccharide chains present on these proteins to bind to, and in many cases to modulate a vast repertoire of proteins (growth factors, cytokines, morphogens, enzymes, structural proteins…).

The four major types of GAGs borne by PGs are heparan sulfate (HS), chondroitin/dermatan sulfate (CS/DS) and keratan sulfate (KS). They are long, linear polysaccharides characterized by a repeating core disaccharide structure comprising an *N*-substituted hexosamine and an uronic acid (a galactose for KS). CS and DS are *N*-actetylgalactosamine (GalNAc)-containing GAGs and essentially differ by the nature of their uronate component: exclusively glucuronic acid (GlcA) for CS, or GlcA and a variable proportion of its C5-epimer iduronic acid (IdoA) for DS. In contrast, HS features glucosamines that can be either *N*-acetylated (GlcNAc) or *N*-sulfated (GlcNS), associated with either GlcA or IdoA residues. These saccharide backbones can be further modified by addition of *O*-sulfate groups: at C-4/C-6 of GalNAc and C-2 of IdoA for CS/DS, and at C-2 of IdoA, C-6 of GlcNAc/GlcNS and occasionally C-3 of GlcNS for HS [5], [6], [7].

GAG structural features, notably extent and patterns of sulfation, largely govern their protein binding and modulating properties. Therefore, the appropriate cell response to signalling proteins is ensured by rapid PG turnover and a highly regulated biosynthesis machinery controlling GAG structure. During PG biogenesis, the synthesis of GAG chains is initiated by the transfer of xylose to specific serine side chains within the protein [1], [7], [8]. This xylosylated protein core is an acceptor for sequential addition of two D-Gal units and D-GlcA and completion of the GAG-protein linkage tetrasaccharide, GlcA(β1–3)Gal(β1–3)Gal(β1–4)Xyl (β1-O)-Ser. The next added saccharide determines the type of newly synthesized GAG chain that will be generated. A GlcNAc saccharide will induce assembly of a HS chain, while a GalNAc saccharide will commit the process toward CS/DS production. The mechanisms regulating this critical step are not fully understood. The presence of acidic and hydrophobic amino acid groups at the vicinity of GAG attachment site, as well as more distant secondary polypeptide structures, has been shown to favour GlcNAc addition [9]. In contrast, C-4 or C-6 sulfation of the Gal saccharides in the tetrasaccharide linker is only seen associated with CS/DS chains [10], [11].

GAG biosynthesis can be initiated in the absence of the core protein, using β-D-xylopyranosides [12], [13], [14]. Xylosides with hydrophobic aglycones are able to penetrate cell membranes, and are used as primers of the core tetrasaccharide for the assembly of soluble GAG chains that are secreted by the cell. The amounts of polysaccharide produced are generally high, nearly quantitative to the level of xyloside present, and chains are usually of reduced length, due to the exhaustion of substrates for the biosynthesis machinery. Most xylosides prime predominately CS/DS synthesis, although it has been observed that HS could be efficiently induced by xylosides with aglycones composed of 2 fused aromatic rings [15], [16]. However, HS priming requires a higher xyloside concentration than what is needed for CS priming.

With regards to their multiple activities, the development of strategies designed to selectively tamper with GAG expression is clearly an attractive prospect to decipher the contribution of specific GAGs in biological functions, or for therapeutic applications. The selective inhibition/knock-down/silencing of GAG biosynthesis enzymes has provided valuable insights into the role of both HS and CS/DS during development, tissue repair or tumour progression [17], [18], [19]. In addition, enzymes, such as heparanase or Sulfs, have been shown to play a critical role in many biological processes through editing modifications of matrix and cell-surface HS and have become prime targets for anti-cancer therapies [20], [21]. Finally, xyloside derivatives have been highlighted as useful tools to interfere with GAG expression. Fluorinated xyloside analogs have been shown to inhibit GAG synthesis [22], and have anti-angiogenic properties [23]. In a different approach, RGD-xyloside conjugates have been synthesized to specifically induce over-expression of free GAG chains localised at the level of α_v_β_3_ integrin expressing cells, and may be useful for the treatment of cardiovascular conditions [24].

Recently, a C-xylopyranoside derivative (C-beta-D-xylopyranoside-2-hydroxy-propane, referred thereafter as C-Xyloside) has been developed to mimic the activity of β-xylosides (that are O-glycosides). This compound showed very similar ability to induce GAG expression as a conventional b-xyloside in cultured dermal fibroblasts [25], but exhibited improved metabolic stability [26], thereby offering interesting perspectives for biological or therapeutic applications. C-Xyloside restored proteoglycan expression in an atrophic human skin model [27] and improved the dermal-epidermal junction in a human reconstructed skin model [28]. Very recently, C-Xyloside treatment of keratinocytes was found to enhance synthesis of GAGs promoting IL-10 dependent cell migration, suggesting a positive action in epithelial repair [29]. In this study, we have aimed at providing the structural basis for these biological activities. For this, we have analysed extensively the effects of C-Xyloside on GAG expression and structure in a 3D model of human reconstituted dermis. Our data indicate that C-Xyloside induces secretion of free GAG chains with distinct structural features (in terms of size, sulfation and molecular organization), but also noticeably affects the fine structural properties of the remaining cell-surface GAGs, leading to altered ligand binding properties.

## Results

### C-Xyloside Strongly Induces the Secretion of Free GAG Chains but does not Affect Expression Levels of Cell-surface GAGs

To analyse the effects of C-Xyloside on GAG biosynthesis, we isolated and purified metabolically labelled GAG chains from RD cultured with [^3^H]glucosamine. Polysaccharides were either recovered from the culture medium (secreted GAGs), or after extraction from RDs with collagenase/Triton/urea (cell-surface and matrix-associated GAGs), yielding four different samples: MedX−/TissueX- and MedX+/TissueX+ corresponding to the culture medium/cell-ECM associated GAGs from untreated or C-Xyloside treated RDs, respectively. These samples were first purified by weak anion-exchange chromatography (DEAE sephacel) followed by analysis of the fractions by scintillation counting.

**Figure 1:**
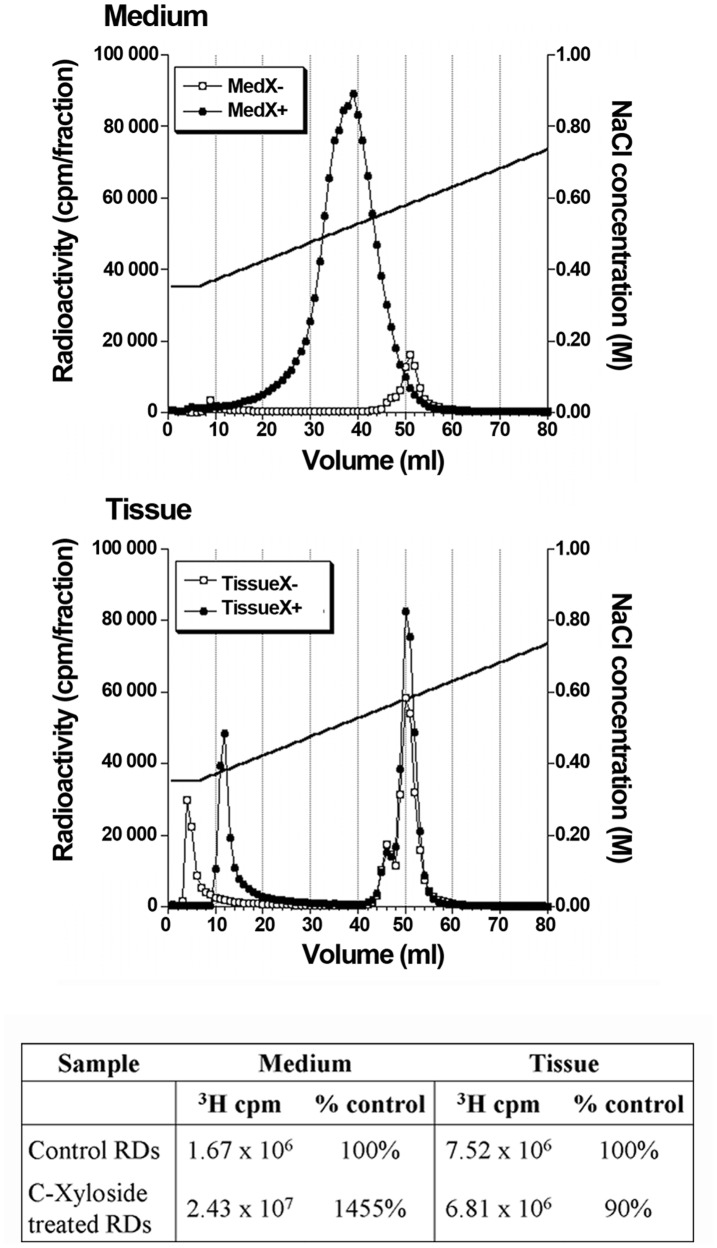
DEAE elution profiles of proteoglycans (PGs) from reconstructed dermis (RDs). ^3^H-labeled medium PGs and tissue-associated PGs treated or not with C-Xyloside (7,5 mM, 48 h) were purified using DEAE ion exchange chromatography. *Black circles*, C-Xyloside treated RDs; *white circles*, control RDs. Incorporated ^3^H radioactivity in medium PGs or in tissue-associated PGs is indicated in the table.

Elution profiles obtained for both cell-associated PG samples TissueX- and TissueX+ were fairly similar, featuring two very close peaks, eluted at ∼560 and ∼580 mM, respectively (Figure 1). In contrast, great discrepancies were noticed between MedX- and MedX+ samples. While the MedX- sample showed a very similar elution profile (2 peaks eluted 560 mM and 580 mM NaCl), the MedX+ sample featured only one broad peak that eluted at a significantly lower NaCl concentration (520 mM) and showed a much higher level of tritium incorporation (15-fold increase compared to the corresponding untreated MedX- sample, see table in Figure 1). This was expected, since xylosides are known to induce secretion of free GAG chains, which would not bind to the DEAE column as tightly as whole PGs featuring multiple polysaccharide chains. However, unlike other xylosides, C-Xyloside did not significantly affect cell/ECM-associated GAG expression levels, tritium incorporation in TissueX- and TissueX+ being comparable (see table in Figure 1). Noteworthy, the presence of xyloside-primed free GAG chains in the TissueX+ fraction could be ruled out, since GAGs from this pool eluted at similar NaCl concentration as the PG-containing TissueX- and MedX- samples. Finally, in addition to PGs, a first peak of tritium at the very beginning of the NaCl gradient was also occasionally detected, corresponding to residual HA that remained bound to the column despite extensive washing with 0.3 M NaCl buffer.

### C-Xyloside Selectively Primes Assembly of CS/DS Chains

**Figure 2:**
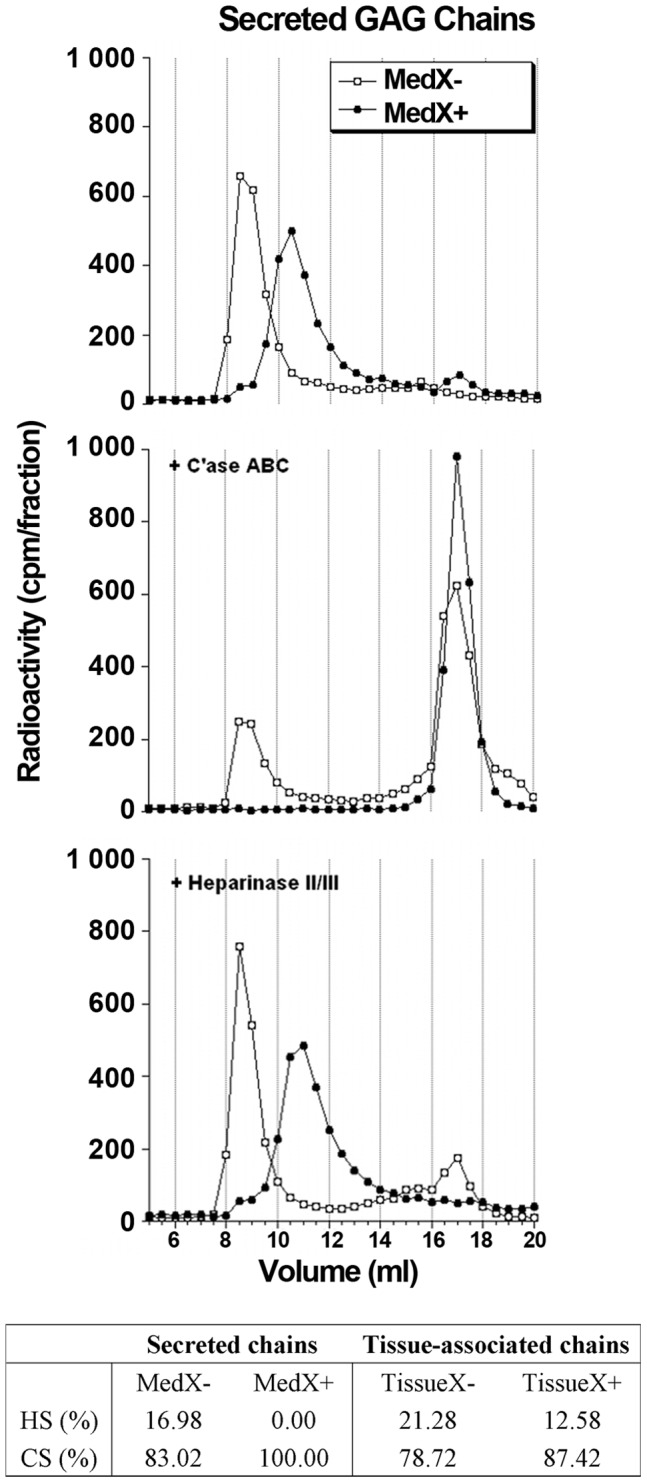
Nature of glycosaminoglycans (GAGs) from RDs. Purified GAGs chains were digested with chondroitinase ABC (C′ase ABC) or heparinase II/III and analysed on a Superdex 75 column. *Black circles*, C-Xyloside treated RDs; *White circles*, control RDs. The ratio of the different synthesized GAGs is shown in the table.

PG-containing fractions were pooled, desalted, then GAG chains were separated from the protein cores by β-elimination and purified again by anion-exchange chromatography. To determine whether C-Xyloside influenced the nature of synthesized GAG chains, we treated the samples with either chondroitinase ABC or a cocktail of heparinases I, II and III and analysed the digestion profiles by gel filtration on a Superdex 75 column. The digestion profile of secreted GAG chains are shown, as an example, in Figure 2. As expected for skin related material, data obtained from control, untreated samples showed that the vast majority of GAG chains produced in the RDs were of the CS/DS type, 83% and 79% of the ^3^H products being eluted in the *V*_t_ column, after chondroitinase ABC digestions of the MedX- and TissueX- samples, respectively (see table in Figure 2). In C-Xyloside treated samples, the predominance of CS/DS was further extended. The most striking effect was observed with the MedX+ sample, which was completely digested by chondroitinase ABC (Figure 2), while it only led to a moderate (∼10%) increase in tissue associated CS/DS *vs* HS content. Sample digestions performed with heparinases yielded complementary data that were in agreement with data obtained with chondroitinase ABC (Figure 2). Altogether, these results confirm that, like many xylosides, C-Xyloside exclusively primes CS/DS chain polymerisation.

### C-Xyloside Primed CS/DS Chains are of Reduced Molecular Size

**Figure 3:**
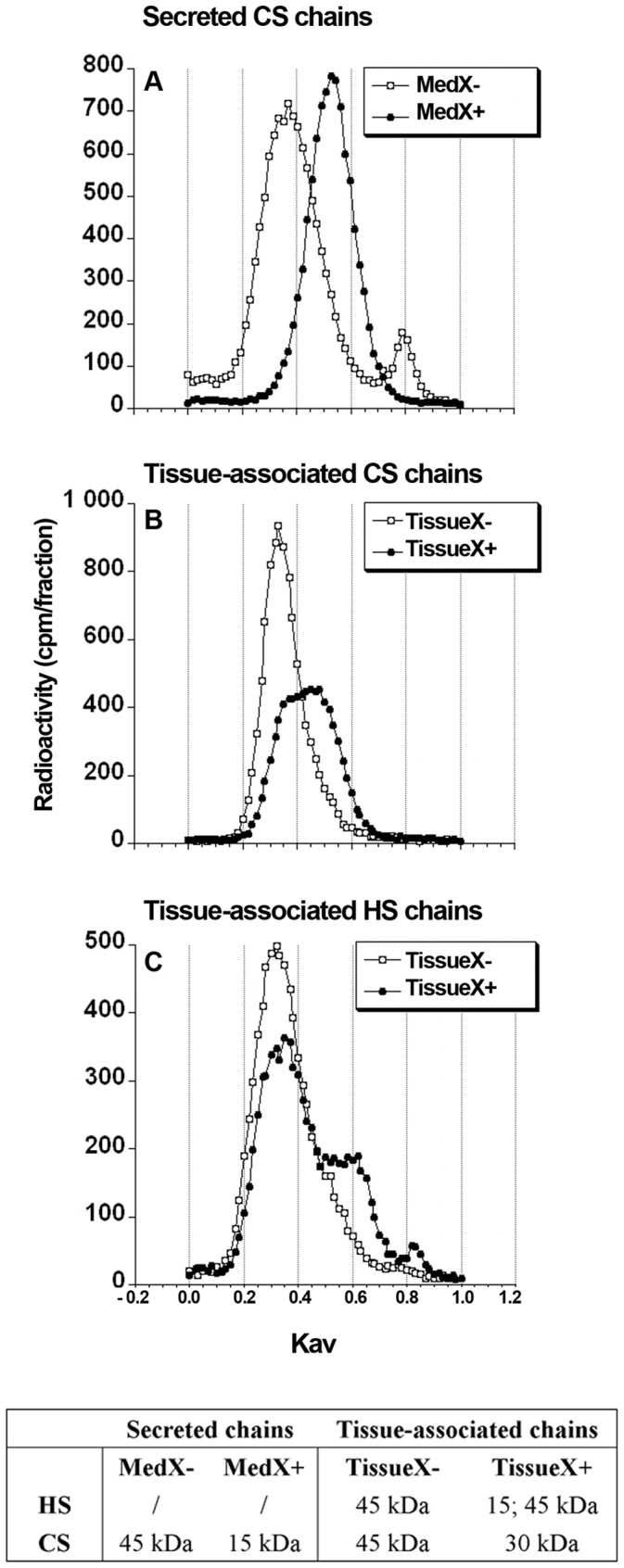
Molecular size determination of the purified HS and CS/DS chains. Molecular size of the HS and CS/DS chains were assessed by gel filtration chromatography on a CL6B column. HS or CS/DS chains were loaded individually on the CL6B column and the fractions collected analyzed by scintillation counting. *Vo* and *Vt* were evaluated using dextran blue and phenol red, respectively. The average molecular masses were estimated using the calibration curve of Wasteson *et al.* [30].

The size distributions of purified GAG chains were analysed by size-exclusion chromatography on a Sepharose CL-6B column, using the previously published calibration chart [30]. Data obtained (see table in Figure 3) showed that both CS and HS chains from control RDs eluted as a single peak, with a *K_av_* of 0.35 (Figure 3, *white circles*) corresponding to a molecular mass of ∼50-kDa. In contrast, treatment with C-Xyloside resulted in a strong reduction of CS chain length in the MedX+ sample, with a peak *K_av_* of 0.53 (Figure 3A, *black circles*), indicating a molecular size of ∼15-kDa only. Tissue-associated GAG chains were also affected, although to a lesser extent. CS chains from the TissueX+ sample had a molecular size of ∼ 30-kDa (*K_av_* = 0.43) (Figure 3B, *black circles*), while two chain sub-populations of ∼15-kDa and ∼50-kDa (*K_av_* = 0,35. and 0.53) were identified for HS (Figure 3C, *black circles*).

### C-Xyloside Affects GAG Chain Composition

**Figure 4:**
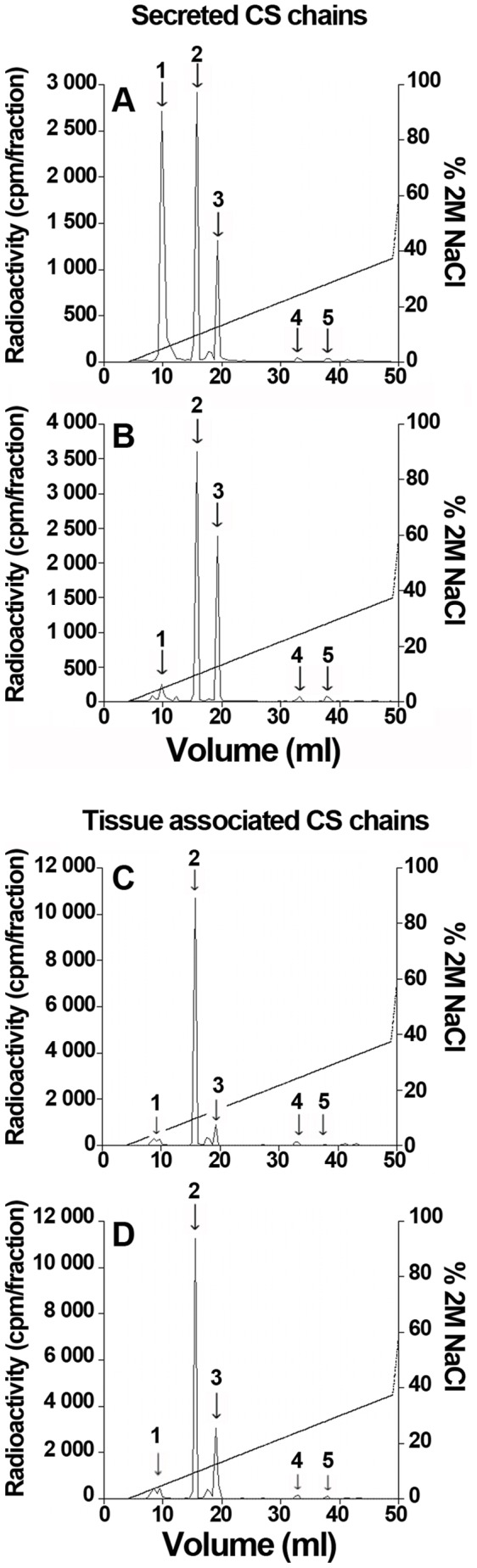
Disaccharide analysis of CS/DS chains from RDs. Secreted or tissue-associated CS chains from control RDs (A,C) and from C-Xyloside treated RDs (B,D) were exhaustively digested with chondroitinase ABC and the digestion products were analysed by SAX-HPLC, using a 45 min linear gradient of 0–0.75 M NaCl. Elution positions of authentic CD/DS disaccharides standards are indicated by arrows.1, ΔDi-0S; 2, ΔDi-4S; 3, ΔDi-6S; 4, ΔDi-2,4S and ΔDi-4,6S; 5, ΔDi-2,6S.

**Figure 5:**
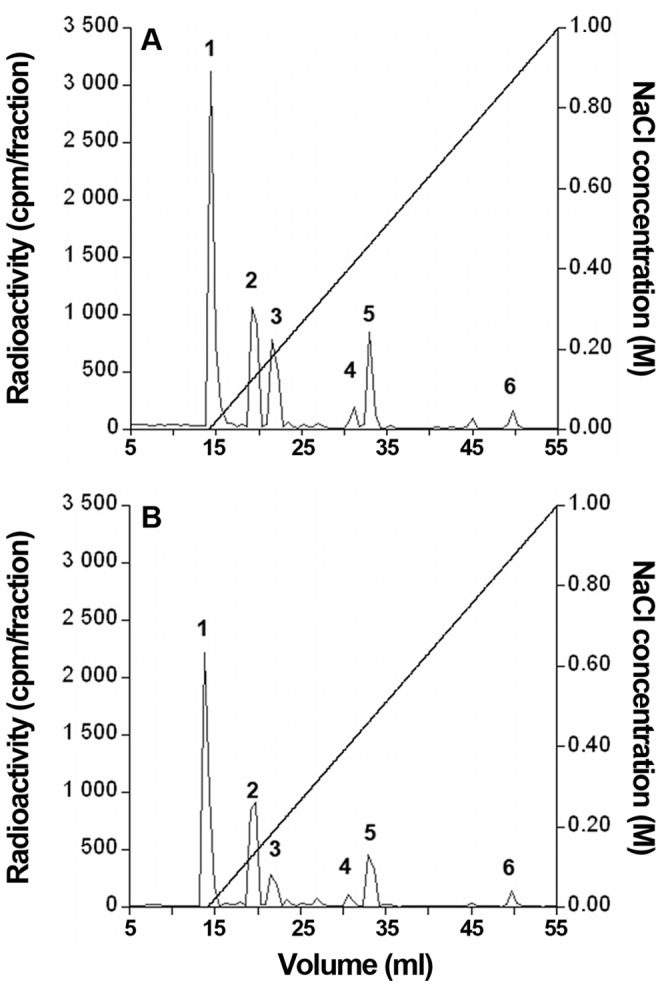
Disaccharide analysis of HS chains from RDs. Tissue-associated HS chains from control RDs (A) and from C-Xyloside treated RDs (B) were exhaustively digested with a combination of heparinases I, II and III and the digestion products were analysed by SAX-HPLC, using a 45 min linear gradient of 0–1 M NaCl. Elution positions of authentic CD/DS disaccharide standards are indicated as follows. *1,* ΔUA-GlcNAc; *2,* ΔUA-GlcNS; *3*, ΔUA-GlcNAc,6S;*4,* ΔUA-GlcNS,6S; *5,* ΔUA,2S-GlcNS; *6*, ΔUA,2S-GlcNS, 6S.

Compositional data were obtained by analysing the disaccharide content of purified GAGs. The CS chains were exhaustively digested to disaccharides using the chondroitinase ABC, the completion of the depolymerisation being verified by gel filtration analysis of the degradation products. Disaccharides were then resolved by SAX-HPLC and identified by comparing the peak elution positions with those of commercial disaccharide standards (Figure 4 and Table 1). Disaccharide analysis of HS chains treated with heparinases I, II and III was achieved similarly (Figure 5 and Table 2).

**Table 1:** Disaccharide composition of CS chains.

Standard peak N°	Disaccharide structure	Total disaccharides			
		*Secreted CS*		*Tissue-associated CS*	
		MedX−	MedX+	TissueX−	TissueX+
**1**	ΔDi-0S	50.3%	6.7%	8.3%	10.5%
**2**	ΔDi-4S	32.6%	53.7%	80.2%	62.0%
**3**	ΔDi-6S	15.1%	35.8%	8.8%	24.8%
**4**	ΔDi-2.4S*ΔDi-4.6S*	1.1%	1.9%	2.2%	1.5%
**5**	ΔDi-2.6S	0.9%	2.0%	0.5%	0.9%
	**4-O-sulfation**	33.7%	55.6%	82.4%	63.5%
	**6-O-sulfation** *	16.0%	37.8%	9.3%	25.7%
	Sulfate/dp2	0.5	1.0	0.9	0.9

* As disaccharides ΔDi-2.4S and ΔDi-4.6S could not be discriminated, corresponding percentage has not been taken into account for the calculation.

Comparison of control and C-Xyloside treated samples revealed major compositional differences. First, CS chains secreted by the C-Xyloside treated RDs featured much higher amounts of mono-sulfated disaccharides (ΔDi-4s + ΔDi-6s = 88%) compared to CS from the MedX- control sample (ΔDi-4s + ΔDi-6s = 47%). Consequently, CS from MedX+ showed a much lower amount of nonsulfated disaccharide ΔDi-0s (7%, *vs* 50% for CS from MedX-). In MedX+ CS, both mono-sulfated species ΔDi-4s and ΔDi-6s increased by relatively similar proportions, indicating no significant selectivity for sulfation on the disaccharide unit. In addition, a small increase was also observed for the minor disulfated disaccharides (ΔDi4,6S/ΔDi2,4S and ΔDi2,6s), again with no species significantly favoured. C-Xyloside therefore induces synthesis of secreted CS chains with much greater charge density (1.0 sulfate/disaccharide for MedX+ CS *vs* 0.5 sulfate/disaccharide for MedX- CS), but does not influence sulfation pattern. Great differences were also observed in tissue-associated CS samples (Table 1). In this case, charge densities of TissueX- and TissueX+ CS were relatively similar (∼0.9 sulfate/disaccharide), the major disaccharide species corresponding to the mono-sulfated disaccharides ΔDi-4s and ΔDi-6s. However, the ratio between these two disaccharides was dramatically shifted upon C-Xyloside treatment. TissueX+ CS showed a much lower ΔDi-4s content compared to the TissueX- control (62% *vs* 80% for TissueX- CS) and conversely, was enriched in ΔDi-6s (25% vs 9% for TissueX- CS).

An effect of C-Xyloside treatment was finally observed on tissue-associated HS disaccharide composition (Table 2). TissueX- HS showed a disaccharide composition relatively similar to that previously published on human skin fibroblast HS [31], whereas TissueX+ HS disaccharides displayed a different pattern (see Table 2). In particular, C-Xyloside treated lead to a strong reduction in *O*-sulfation (33% vs 22% for TissueX- and TissueX+ HS, respectively), mostly 6-O sulfation. Consequently, the overall sulfation content was found to be much lower in HS from C-Xyloside treated RDs.

**Table 2:** Disaccharide composition of HS chains.

Standard peak N°	Disaccharide structure	Total disaccharides	
		TissueX−	TissueX+
**1**	ΔUA-GlcNAc	46.8%	58.5%
**2**	ΔUA-GlcNS	22.4%	21.8%
**3**	ΔUA-GlcNAc.6S	14.6%	5.9%
**4**	ΔUA-GlcNS.6S	3.0%	2.0%
**5**	ΔUA.2S-GlcNS	11.1%	9.6%
**6**	ΔUA.2S-GlcNS.6S	2.2%	2.2%
	**N-sulfation**	36.4%	33.4%
	**2-O-sulfation**	13.3%	11.8%
	**6-O-sulfation**	19.7%	10.1%
	Sulfate/dp2	0.7	0.6

### Molecular Organisation of CS/DS Chains

**Figure 6:**
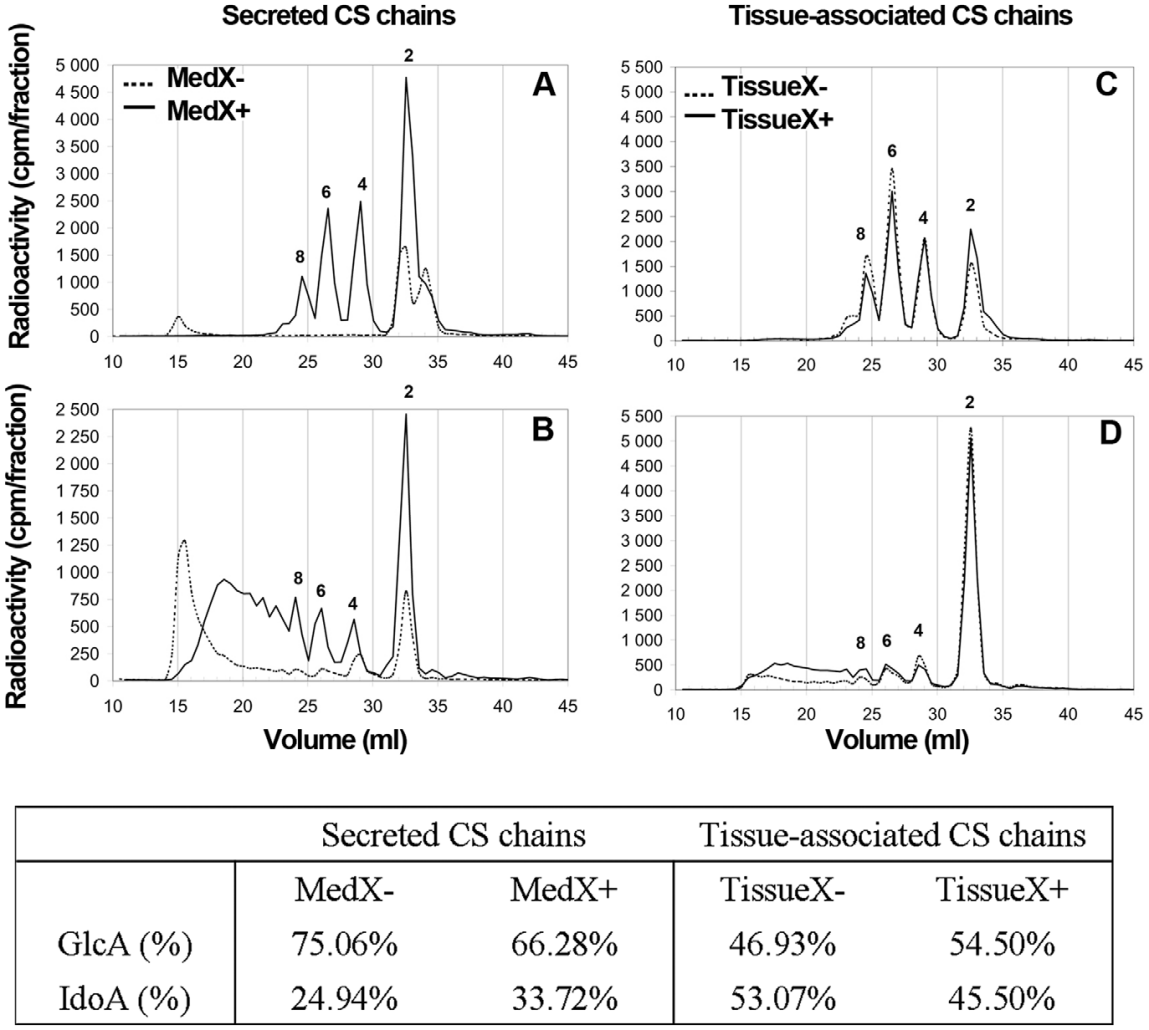
Content and distribution of IdoA residues in CS/DS chains from RDs. Secreted and tissue-associated CS/DS from control RDs (---) and C-Xyloside treated RDs ( ) were degraded by exhaustive treatment with chondroitinase AC-I (**A, C**) or chondroitinase B (**B, D**). Digests were then analysed by size-exclusion chromatography, using two Superdex Peptide columns in series. Elution positions of size-defined oligosaccharides are indicated. The distribution of each oligosaccharide and the ratio of iduronic acid (IdoA) and glucuronic acid (GlcA) are reported in the table.

To analyse the proportion and distribution of iduronic acid (IdoA) and glucuronic acid (GlcA) containing disaccharides, purified CS/DS chains were digested with either chondroitinase ACI or B, and each digest was analysed by gel filtration (Figure 6).

IdoA/GlcA ratios were deduced from the proportion of chondroitinase ACI or chondroitinase B susceptible linkages of our samples (see table in Figure 6). Data showed that C-Xyloside treatment increased the IdoA content of secreted CS/DS chains (25% and 33% for MedX- and MedX+, respectively), but decreased the amount of IdoA in tissue-associated CS/DS (53% and 45% of IdoA for TissueX- and TissueX+, respectively).

Digestion of MedX- CS/DS with chondroitinase B yielded essentially disaccharides, except for a small amount of tetrasaccharides and some large resistant fragments eluting towards *Vo* of the column. These results suggest that within MedX- CS/DS chains, IdoA containing disaccharides are highly segregated in specialised domains, separated by large regions comprising exclusively GlcA units. On the contrary, analysis of MedX+ chondroitinase B digestion showed disaccharides accompanied by a whole range of size defined oligosaccharides (Figure 6B), such pattern suggesting a more regular distribution of IdoA along the chain. Results from the complementary digestion of the samples with chontroitinase ACI were in agreement with these data (Figure 6A). Digestion of MedX- CS/DS into disaccharides (and large undigested fragments) indicated the presence of well apart, exclusively GlcA containing regions, while the presence of many oligosaccharides of intermediate size in the MedX+ digest indicated the presence of alternating IdoA and GlcA units.

Digestion of cell-associated CS (TissueX- and TissueX+) with chondroitinase ACI or B, resulted in the formation of oligosaccharides corresponding to di-, tetra-, hexa-, octa-, and decasaccharides, suggesting a mixed distribution of the GlcA and IdoA containing units along the polysaccharide chains, both in the control RDs and in the C-Xyloside treated RDs.

### C-Xyloside Affects Cell-associated CS Biological Properties

**Figure 7:**
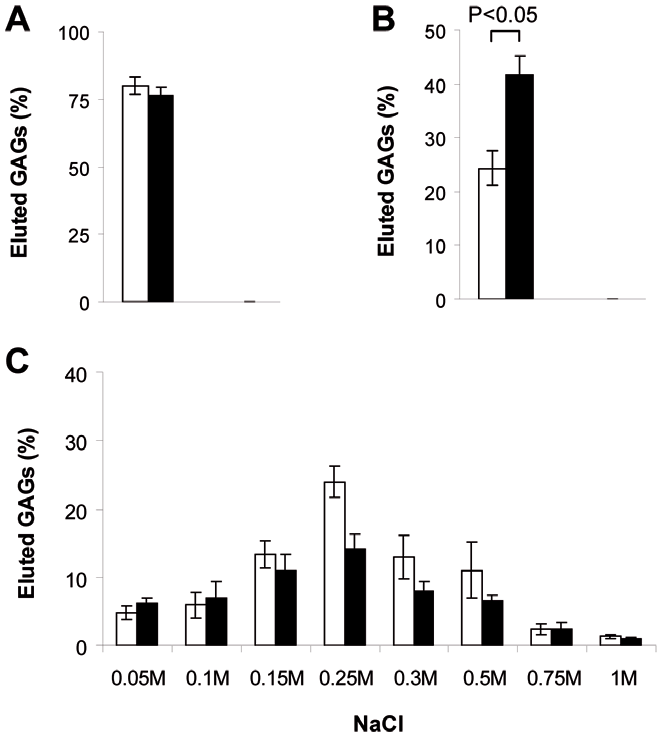
Binding of HGF to CS/DS chains from RDs. Secreted (**A**) and tissue-associated (**B**) CS/DS from control RDs (white bars) and C-Xyloside treated RDs (Black bars) were incubated with HGF and drawn through a nitrocellulose filter. Amounts of unbound GAG was assessed by scintillation counting of the wash-through and expressed as a percentage of total material (10,000 cpm). For tissue-associated GAGs, bound material was then eluted by sequential washing of the filter with increasing NaCl concentrations (**C**). Results represent means of three independent experiments. Error bars indicate sd and statistical significance (in B) was calculated with a Student’s t test.

We then assessed the consequences of C-Xyloside induced structural changes on the ability of CS to bind to HGF. HGF binding properties of RD GAGs were analysed using a filter binding assay. To do so, the growth factor (1 µg) was incubated with 10 000 cpm of each GAG sample, then drawn through a nitrocellulose membrane. Free GAG chains were recovered in the wash-through (and buffer rinses), while HGF bound material was step-eluted from the membrane with increasing NaCl concentrations. Secreted CS/DS did not significantly bind to HGF, regardless of C-Xyloside treatment (Figure 7A), whereas a great proportion of tissue-associated CS/DS was retained on the filter (Figure 7B). Interestingly, C-Xyloside treatment led to a significant increase in the amount of radiolabelled material that eluted straight through the filter (70% increase compared to the TissueX- control sample). This suggests that the structural modifications occurring in tissue-associated CS upon C-Xyloside treatment impairs recognition of and binding to HGF. Bound material from both TissueX- and TissueX+ samples were eluted from the filter at a similar ionic strength (Figure 7C). Finally, a proportion of cell-associated HS was also found to bind to HGF, but no differences in the amount of bound material or in the ionic strength required for elution could be noticed between TissueX- and TissueX+ samples (data not shown).

## Discussion

GAGs are polysaccharides that are critically involved in many biological processes. However, study of these molecules remains extremely difficult because of their inherent heterogeneity and structural complexity. This is clearly exemplified in skin, where GAGs are believed to play important, yet not fully defined roles. GAGs are indeed major components of dermal ECM and participate in tissue cohesiveness and hydration. Through their ability to bind to and modulate the activity of a number of growth factors, GAGs are also involved in cell adhesion and migration, as well as skin organogenesis and wound healing. The structure and integrity of GAGs is therefore essential for skin homeostasis and regeneration, leaving open the question of a potential role of GAGs in the different skin stem cell compartments (including epidermal and dermal stem cells) known to be involved in skin homeostasis. The consequences of skin ageing on GAGs remain poorly understood. However, increasing evidence suggest that age-related alteration of the dermal connective tissue may involve a remodeling of GAG expression and structure [32], [33], [34]. In addition, a recent publication reports that senescence of dermal stem cells occurs during the aging process and affects their functions [35], highlighting the need to investigate the possible connection between GAGs, stem cells and aging in the near future. In that context, it would be interesting to focus on the role of growth factors and certain chemokines/cytokines (such as IL7, Fractalkine, G-CSF) that bind to GAGs and are known to be secreted by keratinocytes during the wound healing process [36]. These factors appear like potential candidates to understand the mechanisms involved in the (mis)communication between the different stem cell compartments along human lifetime. GAGs may therefore constitute attractive targets in cosmetology, wound healing, or for the development of new therapeutic agents against skin disease.

In this context, xylosides could provide an important orthogonal method to selectively alter the expression and structure of the GAGs present at cell surfaces, although their effects on the polysaccharide fine structural features remains poorly documented. On this basis, we have recently developed a C-Xyloside, that presents the same GAG-priming activity as classical β-xylosides, but exhibits improved chemical stability and therefore shows greater potential for future *in vivo*/therapeutic applications [25], [26]. During our previous studies, GAG-inducing activity of C-xyloside was confirmed and was shown to be very similar as that of a control β-xyloside in cultured human dermal fibroblasts [25]. In addition, we showed using a number of models that C-Xyloside restored compromised proteoglycan expression in atrophic dermis [27], improved dermal-epidermal junction [28] and promoted epidermal keratinocyte migration [29]. However, understanding further the underlying mechanisms would require first the precise characterisation of GAG structural alterations induced by such compound. Here, we have investigated these aspects in the line of our previous study on monolayer-cultured dermal fibroblasts [25]. However, to improve further the relevance of our data, we have used here a reconstituted human skin dermis model, to provide an extracellular environment as close as possible to the physiological one, while limiting the study to this single cell type.

Our results first confirmed the previously observed induction of GAG synthesis by C-Xyloside [25]. We found that C-Xyloside treatment of RDs resulted in a 15 fold increase of GAGs found in the extracellular medium, these GAGs being exclusively CS/DS chains (Table 3). Such results are in agreement with a number of studies, which showed that most xylosides specifically primed assembly of free CS/DS chains, produced in high amounts and exported outside the cell [12], [13], [14]. Likewise, we also found that C-xyloside primed CS/DS chains were of significantly shorter size, likely as a result of the GAG biosynthesis burden. However, and unlike some findings with other xylosides, C-Xyloside did not lead to a significant reduction in tritium incorporation within tissue-associated GAGs, suggesting no effect on the expression of GAGs borne by proteoglycans. Disaccharide analysis revealed effects of C-Xyloside on both xyloside-primed and proteoglycan-associated GAG chains. C-Xyloside primed CS chains found in the extracellular medium showed an increased level of overall sulfation, mainly due to higher amounts of monosulfated species to the detriment of the unsulfated one. However, the nature of sulfation remained unchanged. Interestingly, the polysaccharide organisation in CS or DS domains was also affected. Chains from untreated RDs were mainly of the CS type, with IdoA-containing disaccharides well segregated into distant domains within the polysaccharide. In contrast, C-Xyloside treatment resulted in a more regular distribution of these disaccharides along the chain (Table 3). Polysaccharides borne by proteoglycans present at the cell surface and in the surrounding ECM were also structurally affected by C-Xyloside, although in very different ways. C-Xyloside treatment did not affect the polysaccharide overall charge, and IdoA distribution. However, it modified the nature of sulfation on these chains, the main difference being a reduction of ∼25% of the 4-O-sulfated disaccharides compensated by an equivalent increase of 6-O-sulfated disaccharides. Although unable to induce HS synthesis, C-Xyloside also affected the structure of PG-associated HS. Treatment with C-Xyloside resulted in a substantial reduction in O-sulfation, mostly 6-O-sulfation (Table 2). Interestingly, the most significant effect was a reduction of (ΔUA-GlcNAc,6S) disaccharide and the consequent increase in (ΔUA-GlcNAc) disaccharide. Such disaccharide is predominantly found in HS S-domain flanking regions. C-Xyloside may therefore induce directed alterations of 6-O-sulfation patterns within these areas, which could lead to changes in defined biological functions.

**Table 3:** Summary of C-Xyloside effects on GAG expression and structure.

Features	Effects of C-Xyloside treatment	
	*Secreted GAGs*	*Tissue-associated GAGs*
Total GAG amount	1 → 15	No change
Ratio HS : CS/DS	1∶5 → no more HS	1∶4 → 1∶7
GAG chain size	CS/DS: 45 → 15 kDa	CS/DS : 45 → 30 kDa HS : 45 → 15 and 45 kDa
CS/DS global charge (sulfate/dp2)	∼0.5→ ∼1	No change (∼0.9)
Ratio CS/DS 4-O−/6-O-sulfation	∼2∶1→ ∼2∶1	∼7∶1→ ∼2.5∶1
HS N−/O-sulfation	–	N–S : no change; O–S: −11%
CS/DS GlcA : IdoA distribution	Segregated → more evenly distributed	No change
HGF binding (CS/DS)	No change	−26% of binding

Altogether, our data indicate that C-Xyloside has numerous and complex effects on GAGs. C-Xyloside does not simply induce an upregulation of CS/DS production, but also affects GAG structure, including subtle features such as altered sulfation profiles. Interestingly, these modifications differ, depending on whether the polysaccharide chains are primed by the xyloside or attached to a protein core. This suggests that C-Xyloside may have some regulatory function on GAG biosynthesis machinery. Effects of C-Xyloside on proteoglycans have been previously reported. Syndecan-1, Syndecan-4 and Perlecan depleted expression in an atrophic skin model was restored to normal level by treatment with C-Xyloside [27]. However, our study provides the first evidence of a regulatory effect on the activity of GAG biosynthesis sulfotransferases. Further work will be needed to determine whether this effect is due to a direct modulation of biosynthesis enzyme expression, or to the burdened biosynthesis machinery, leading to enzyme titration and assembly on PGs of distinct saccharide motifs.

As GAG structural and functional properties are closely intertwined, we foresaw that C-Xyloside induced structural modifications of the polysaccharide would have consequences on its activity. We therefore tested the ability of GAGs from untreated and C-Xyloside treated RDs to bind HGF, a growth factor that can interact with both HS and CS/DS. HGF is a multifunctional growth factor promoting motility and proliferation of many different cell types, mainly of mesenchymal origin [37]. In skin, HGF is mainly produced by dermal fibroblasts and has a paracrine action on epidermal keratinocytes, thus contributing to important dermis/epidermis crosstalk [38]. Our results showed that the structural modifications of cell-associated CS/DS induced by C-Xyloside greatly reduced their ability to interact with HGF. Although the functional consequences of this loss of binding activity have not been examined yet, one attractive hypothesis is that C-Xyloside may modify these dermis/epidermis crosstalks by facilitating diffusion of HGF towards keratinocytes. However, much work will be needed to completely decipher the mechanisms involved, in a biological system as complex as the skin, and with other cytokines/growth factors likely to be affected by these alterations.

From a structural point of view, our data provide new information on GAG/HGF interaction. GAG structural requirements for binding to HGF remains unclear. Heparin and HS display the highest affinity for the growth factor, with a direct correlation between binding and charge content but no definite requirements regarding sulfation positions [39], [40], [41]. Accordingly, our results show that the C-Xyloside-induced structural changes in HS, which are restricted to the relatively low sulfated S-domain flanking regions, do not affect HGF binding. For DS, a comparative analysis of mammalian DS and highly sulfated DS isolated from marine tunicates *Ascidia nigra* showed no specific O-sulfation requirements for binding to HGF but, unlike HS/heparin, no significant correlation between overall sulfation and binding affinity [40]. This study also emphasized the critical role played by DS IdoA units, which could compensate for a relatively low level of overall sulfation by increasing the polysaccharide chain flexibility and facilitating protein-saccharide contacts [40]. Our data is in partial agreement with these conclusions, since secreted GAG chains which feature lower IdoA content than their tissue-associated counterparts (Figure 6) failed to promote binding to HGF (Figure 7A), and the significant increase of overall sulfation induced by C-Xyloside did not suffice to restore binding. However, our results also show a requirement for 4-O-sulfation, as C-Xyloside 4-O-sulfation reduction on tissue-associated CS/DS resulted in impaired HGF binding, despite increased 6-O-sulfation (Figure 7B). This is in apparent contradiction with the results obtained on Ascidian DS, which binds to HGF but lacks 4-O-sulfates. One likely explanation would be that this DS species is particularly enriched in IdoA (nearly 100% of uronates, compared to ∼50% in our dermal CS/DS preparations), which may compensate for the absence of 4-O-sulfates. Our data thus support further the existence of a critical interplay between DS sulfation and iduronate content [40], [42]. Moreover, they highlight the importance of 4-O-sulfates for the interaction, which may become a prerequisite for the binding of HGF to GAGs with low levels of IdoA.

In conclusion, this study supplies missing structural information that should help understanding the exact mechanisms underlying the beneficial effects of C-Xyloside on dermis/epidermis homeostasis and regeneration. In a more general perspective, this study delivers a detailed and unprecedented survey of the effect of a xyloside on GAG expression and fine structure and provides new insights into the activity mechanisms of xylosides. Much work would be needed to determine the whole repertoire of C-Xyloside activities and applications. C-xyloside will most likely affect differently GAG binding properties for its various ligands. C-Xyloside effects on GAG structure may also vary from one cell type to another, and may be highly dependent on the amount of GAGs, or the proportion of CS/DS versus HS, naturally expressed by these cells. Finally, although C-xyloside overall effect on GAG expression was comparable to that of a conventional β-xyloside, we cannot exclude differences in the way these compounds alter GAG fine structure. Likewise, variations in xyloside structure (particularly of their aglycone moiety) may trigger different activities. However, this study shows for the first time that the GAG modifying activities of these molecules are not restricted to xyloside-primed GAG chains, and demonstrates that these modifications lead to a fine tuning of GAG structure. Finally, as exemplified with the analysis of HGF binding activity, we bring out the use of xylosides as an efficient strategy to induce GAG structural modifications and decipher fine structure/activity relationships involved in biological functions of these polysaccharides, as well as for potential corrective applications.

## Materials and Methods

### Ethics

Skin fibroblasts were recovered from anonymous breast surgical waste. Samples were obtained for research purposes and donors gave their written informed consent as is required by French Bioethics Law of 2004 (article L1245-2). This law exempts this research from requiring formal ethical approval.

### Materials

D-[1-^3^H] Glucosamine (sp. radioactivity 20–45 Ci/mmol) was obtained from Perkin Elmer. Scintillation mixture (Optiphase HiSafe 3) was obtained from PerkinElmer Life Sciences. C-β-D-xylopyranoside-2-hydroxy-propane (C-Xyloside, Pro-xylane™) was obtained from L’Oréal Research Laboratories (Clichy, France), as previously described [26]. Chondroitinase ABC (*Proteus vulgaris;* EC 4.2.2.4) and chondroitinase B (*Flavobacterium heparinum;* EC 4.2.2.19*)* were purchased from Sigma-Aldrich Co, chondroitinase AC I (*Flavobacterium heparinum;* EC 4.2.2.5*)* from Seikagaku Kogyo Co. (Tokyo, Japan); Heparinase I, II and III were from Grampian enzymes (Orkney, UK). Complete™ protease inhibitor cocktail was from Roche diagnostics (Meylan, France).

4,5-unsaturated CS/DS disaccharide standards (ΔUA-GalNAc (ΔDi-0S), ΔUA-GalNAc4S (ΔDi-4S), ΔUA-GalNAc6S (ΔDi-6S), ΔUA2S-GalNAc (ΔDi-2S), ΔUA2S-GalNAc4S (ΔDi-2,4S), ΔUA2S-GalNAc6S (ΔDi-2,6S), ΔUA-GalNAc4S6S (ΔDi-4,6S), ΔUA2SGalNAc4S6S (ΔDi-2,4,6S)) were from Iduron (Manchester, UK). All other chemicals were from Sigma or of equivalent commercial grade.

### Cell Culture and Metabolic Labelling

Normal human breast skin was obtained after written informed consent from healthy subjects following plastic mammary reduction and according to the principles expressed in the Declaration of Helsinki. Dermal fibroblasts were then isolated from skin samples as previously described [43] and cultured in DMEM medium (Perbio, Brebieres, France) supplemented with 10% newborn calf serum (Thermo Scientific Hyclone, Illkirch, France) with antibiotics at 37°C with 5% CO_2_. At the 6^th^ passage, cells were seeded at a concentration of 250 000 cells per cm^2^ onto chemically cross-linked collagen sponges (obtained from L’Oréal Research Laboratories, Clichy, France) to produce the reconstructed dermis [44]. Cells were cultured for 14 days in DMEM medium (Perbio) supplemented with 10% newborn calf serum (Hyclone) with antibiotics (Sigma) and 0.5 mM of ascorbic acid −2-phosphate (Sigma) at 37°C with 5% CO_2_. At Day 14, reconstructed dermis (RD) were incubated in fresh medium supplemented or not with C-Xyloside for 48 h, then cells were metabolically radiolabelled by adding 10 µCi/ml of D-[1-^3^H] Glucosamine for another 48 h. On cell monolayers, optimal effect on GAG expression was achieved from 1 mM and above of C-Xyloside [27]. Here, RDs were treated with 7.5 mM C-Xyloside to circumvent possible reduction in efficacy resulting from hindered diffusion/cell accessibility within the tissue.

### Preparation of Purified GAG Chains

Following metabolic labelling, the medium was removed and RDs were rinsed twice with PBS. Medium and washes were pooled and stored at −20°C. RDs were incubated with collagenase (2 mg/ml in PBS, 5 mM CaCl_2_) at 37°C for 2 h and centrifuged for 5 minutes at 3000 rpm. Supernatants were recovered and cells and ECM were incubated with a Triton X-100 extracting solution (20 mM phosphate pH 6.5, 0.25 M NaCl, supplemented with 1% Triton X-100 (v/v), 10 mg/ml bovine serum albumin and Complete™ protease inhibitor cocktail) for 30 minutes at 4°C, under stirring. Extracts were centrifuged for 10 minutes at 15 000 rpm, supernatants were retrieved and pellets were incubated overnight at 4°C with urea-extract buffer (20 mM phosphate pH 6.5, 0.25 M NaCl, 6 M urea, 1% Triton X-100 (v/v), 10 mg/ml bovine serum albumin, Complete™ protease inhibitor cocktail). The resulting extracts were centrifuged for 10 minutes at 15 000 rpm, supernatants were recovered and pooled with the two previous supernatant fractions.

The medium or tissue samples were applied to an ion-exchange DEAE-Sephacel column (1×10 cm). The column was first washed with 20 mM Phosphate pH6.5, 0.3 M NaCl, to remove contaminating proteins and hyaluronic acid, then PGs were resolved on a linear 330 min gradient of 0.35–0.75 M NaCl in 20 mM Phosphate pH 6.5, at a flow rate of 0.250 ml/min. Fractions of 1 ml were collected and aliquots of 50 µl were removed for scintillation counting (Packard Tri-Carb 2100 TR β-counter). Fractions corresponding to detected radioactive peaks were pooled and freeze-dried. Samples were solubilised in distilled water and desalted on a PD-10 column (GE Healthcare), equilibrated in distilled water.

GAG chains were then released from the core protein by β-elimination, as previously described [45]. Samples were incubated in 0.05 M NaOH, 1 M sodium borohydride and incubated for 48 h at 45°C. The reaction was stopped by adding glacial acetic acid and pH was neutralised using NaOH.

### Molecular Sizing of GAG Chains

Purified GAG chains were analysed by size-exclusion chromatography, using a Sepharose CL-6B column (1 cm diameter×120 cm length) equilibrated in PBS, at a flow rate of 4 ml/h. Fractions (1 ml) were collected and ^3^H content was monitored by scintillation counting. Fraction numbers were normalised to *K_av_* values, calculated from the *V_O_* and *V*_t_ values for dextran blue and phenol red respectively, which were added to the sample prior to column loading. Molecular weight of GAG chains was then calculated from *K_av_* values using the Wasteson calibration [30]. Samples treated with either chondroitinase AC or chondroitinase B were also analysed by CL-6B gel chromatography, following the same protocol.

### Disaccharide Analysis

Purified [^3^H]-CS/DS chains (∼20,000 cpm) were exhaustively digested to disaccharides by adding 500 mU of chondroitinase ABC in 50 mM Tris-HCl pH 7,5, 50 mM NaCl, 2 mM CaCl_2_, 0.01% (w/v) bovine serum albumin, at 37°C for 24 h. The enzyme was denatured by boiling the samples for 5 min and precipitated by centrifugation at 13 000 rpm. The completion of the reaction was confirmed by gel chromatography analysis, using two Superdex Peptide 10/300GL columns in series equilibrated in 1 mM KH_2_PO_4_, 3 mM Na_2_HPO_4_,2H_2_O, 350 mM NaCl pH 7,4, at a flow rate of 0.5 ml/min. Generated disaccharides were subsequently reduced by incubation in 0.1 M NaBH_4_, 10 mM NaOH for 2 h at room temperature. Remaining NaBH_4_ was hydrolysed by acidification with 2 M acetic acid and pH was neutralised with NaOH. Samples were then applied to a Propac PA1 strong-anion exchange column (4×250 mm; Dionex) equilibrated in water pH 3.5. After a wash with water pH 3.5, disaccharides were resolved over a linear gradient of 0–0.75 M NaCl, pH 3.5, at a flow rate of 1 ml/min. Fractions (0.5 ml) were collected and analysed by scintillation counting. Disaccharides peaks were identified by comparison with the elution positions of CD/DS disaccharides standards.

Purified [^3^H]-HS chains (∼50 000 cpm) were exhaustively digested to disaccharides after successive addition of 10 mU of heparinase I in 100 mM sodium acetate pH 7.1, 0.5 mM calcium acetate at 30°C for 24 h, then heparinase II and heparinase III (10 mU of each) at 37°C for 24 h. Digests were boiled and centrifuged to inactivate and remove enzymes. Like CS/DS chains, the completion of the reaction was confirmed by gel chromatography using the twinned Superdex Peptide columns. Samples were applied to the Propac PA1 equilibrated in water pH 3.5 and resolved on a linear NaCl gradient, 0–1 M NaCl, pH 3.5 over 45 min at a flow rate of 1 ml/min. Fractions (0.6 ml) were collected and analysed by scintillation counting. Disaccharides peaks were identified by comparing the elution positions of HS disaccharides standards.

### Determination of the Glucuronate: Iduronate Ratio

[^3^H] purified GAG chains (50 000 cpm) were exhaustively digested with either chondroitinase ACI (0.25 units/ml in 33 mM Tris-HCl, 33 mM sodium acetate, 0.008% (w/v) bovine serum albumin, pH 7.3) or chondroitinase B (12.5 units/ml in 20 mM Tris-HCl, 50 mM NaCl, 4 mM CaCl2, 0.01% (w/v) bovine serum albumine pH 7.4) at 37°C for 24 h. Digests were then boiled and centrifuged. Supernatants were applied to two Superdex Peptide 10/300GL columns in series equilibrated with 0.35 M NaCl and run at a flow rate of 0.5 ml/min. Fractions of 0.5 ml were collected and counted for radioactivity. The percentage of galactosaminyl bonds cleaved by each enzyme was calculated from the distribution of ^3^H radiolabeled peaks, relative to the total eluted ^3^H radiolabel, using the standard algorithm. GlcA content was therefore calculated from chondroitinase ACI digestion profile as follow: %GlcA =  Σ (*P_n_/n*), *P_n_* being the percentage of total ^3^H found in a peak corresponding to a *n* disaccharide fragment, as determined by the elution position.

### Filter Binding Assay

Filter binding analysis was performed as previously described [46]. Briefly, [^3^H] GAG chains (10 000 cpm) were incubated with 1 µg of HGF in 200 µl of 25 mM Tris-HCl, pH7.5 for 45 min at room temperature. Samples were then drawn through buffer equilibrated nitrocellulose filters, using a vacuum manifold (Millipore). Filters were washed with 2×500 µl Tris buffer then collected and pooled with the sample wash-through. Bound GAGs were step-eluted by washing the filters with 2×500 µl of Tris buffer containing increasing NaCl concentrations. The collected NaCl washes (as well as the wash-through) were analysed by scintillation counting.

## References

[1] Sarrazin S, Lamanna WC, Esko JD (2011) Heparan sulfate proteoglycans. Cold Spring Harb Perspect Biol 3. doi: 10.1101/cshperspect.a004952. 10.1101/cshperspect.a004952 PMC3119907 21690215

[2] Spillmann D (2001) Heparan sulfate: anchor for viral intruders? Biochimie 83: 811–817. 11530214 10.1016/s0300-9084(01)01290-1

[3] Delehedde M, Allain F, Payne SJ, Borgo R, Vampouille C, et al (2002) Proteoglycans in Inflammation. Curr Med Chem 1: 89–102.

[4] Sasisekharan R, Shriver Z, Venkataraman G, Narayanasami U (2002) Roles of heparan-sulphate glycosaminoglycans in cancer. Nat Rev Cancer 2: 521–528. 12094238 10.1038/nrc842

[5] Esko JD, Kimata K, Lindahl U (2009) Proteoglycans and Sulfated Glycosaminoglycans. In: Varki A CR, Esko JD, Freeze H, Hart G, Marth J, editor. Essentials of Glycobiology 2nd edition. Cold Spring Harbor Laboratory Press. 20301236

[6] Lindahl U, Li JP (2009) Interactions between heparan sulfate and proteins-design and functional implications. Int Rev Cell Mol Biol 276: 105–159. 19584012 10.1016/S1937-6448(09)76003-4

[7] Zhang L (2010) Glycosaminoglycan (GAG) biosynthesis and GAG-binding proteins. Prog Mol Biol Transl Sci 93: 1–17. 20807638 10.1016/S1877-1173(10)93001-9

[8] Esko JD, Lindahl U (2001) Molecular diversity of heparan sulfate. J Clin Invest 108: 169–173. 11457867 10.1172/JCI13530 PMC203033

[9] Zhang L, Esko JD (1994) Amino acid determinants that drive heparan sulfate assembly in a proteoglycan. J Biol Chem 269: 19295–19299. 8034692

[10] Ueno M, Yamada S, Zako M, Bernfield M, Sugahara K (2001) Structural characterization of heparan sulfate and chondroitin sulfate of syndecan-1 purified from normal murine mammary gland epithelial cells. Common phosphorylation of xylose and differential sulfation of galactose in the protein linkage region tetrasaccharide sequence. J Biol Chem 276: 29134–29140. 11384972 10.1074/jbc.M102089200

[11] Gulberti S, Lattard V, Fondeur M, Jacquinet JC, Mulliert G, et al (2005) Phosphorylation and sulfation of oligosaccharide substrates critically influence the activity of human beta1,4-galactosyltransferase 7 (GalT-I) and beta1,3-glucuronosyltransferase I (GlcAT-I) involved in the biosynthesis of the glycosaminoglycan-protein linkage region of proteoglycans. J Biol Chem 280: 1417–1425. 15522873 10.1074/jbc.M411552200

[12] Kuberan B, Ethirajan M, Victor XV, Tran V, Nguyen K, et al (2008) “Click” xylosides initiate glycosaminoglycan biosynthesis in a mammalian cell line. Chembiochem 9: 198–200. 18085541 10.1002/cbic.200700494

[13] Lugemwa FN, Sarkar AK, Esko JD (1996) Unusual beta-D-xylosides that prime glycosaminoglycans in animal cells. J Biol Chem 271: 19159–19165. 8702593 10.1074/jbc.271.32.19159

[14] Prydz K, Vuong TT, Kolset SO (2009) Glycosaminoglycan secretion in xyloside treated polarized human colon carcinoma Caco-2 cells. Glycoconj J 26: 1117–1124. 19252982 10.1007/s10719-009-9232-2

[15] Fritz TA, Lugemwa FN, Sarkar AK, Esko JD (1994) Biosynthesis of heparan sulfate on beta-D-xylosides depends on aglycone structure. J Biol Chem 269: 300–307. 8276811

[16] Lugemwa FN, Esko JD (1991) Estradiol beta-D-xyloside, an efficient primer for heparan sulfate biosynthesis. J Biol Chem 266: 6674–6677. 2016281

[17] Bulow HE, Hobert O (2006) The molecular diversity of glycosaminoglycans shapes animal development. Annu Rev Cell Dev Biol 22: 375–407. 16805665 10.1146/annurev.cellbio.22.010605.093433

[18] Forsberg E, Kjellen L (2001) Heparan sulfate: lessons from knockout mice. J Clin Invest 108: 175–180. 11457868 10.1172/JCI13561 PMC203035

[19] Kusche-Gullberg M, Kjellen L (2003) Sulfotransferases in glycosaminoglycan biosynthesis. Curr Opin Struct Biol 13: 605–611. 14568616 10.1016/j.sbi.2003.08.002

[20] Miao HQ, Liu H, Navarro E, Kussie P, Zhu Z (2006) Development of heparanase inhibitors for anti-cancer therapy. Curr Med Chem 13: 2101–2111. 16918340 10.2174/092986706777935230

[21] Rosen SD, Lemjabbar-Alaoui H (2010) Sulf-2: an extracellular modulator of cell signaling and a cancer target candidate. Expert Opin Ther Targets 14: 935–949. 20629619 10.1517/14728222.2010.504718 PMC3126665

[22] Garud DR, Tran VM, Victor XV, Koketsu M, Kuberan B (2008) Inhibition of heparan sulfate and chondroitin sulfate proteoglycan biosynthesis. J Biol Chem 283: 28881–28887. 18708345 10.1074/jbc.M805939200 PMC2570865

[23] Raman K, Ninomiya M, Nguyen TK, Tsuzuki Y, Koketsu M, et al (2011) Novel glycosaminoglycan biosynthetic inhibitors affect tumor-associated angiogenesis. Biochem Biophys Res Commun 404: 86–89. 21094131 10.1016/j.bbrc.2010.11.069 PMC3031167

[24] Tran VM, Victor XV, Yockman JW, Kuberan B (2010) RGD-xyloside conjugates prime glycosaminoglycans. Glycoconj J 27: 625–633. 20717719 10.1007/s10719-010-9306-1 PMC2975667

[25] Pineau N, Carrino DA, Caplan AI, Breton L (2011) Biological evaluation of a new C-xylopyranoside derivative (C-Xyloside) and its role in glycosaminoglycan biosynthesis. Eur J Dermatol 21: 359–370. 21609902 10.1684/ejd.2011.1340

[26] Cavezza A, Boulle C, Gueguiniat A, Pichaud P, Trouille S, et al (2009) Synthesis of Pro-Xylane: a new biologically active C-glycoside in aqueous media. Bioorg Med Chem Lett 19: 845–849. 19135365 10.1016/j.bmcl.2008.12.037

[27] Pineau N, Bernerd F, Cavezza A, Dalko-Csiba M, Breton L (2008) A new C-xylopyranoside derivative induces skin expression of glycosaminoglycans and heparan sulphate proteoglycans. Eur J Dermatol 18: 36–40. 18086587 10.1684/ejd.2008.0308

[28] Sok J, Pineau N, Dalko-Csiba M, Breton L, Bernerd F (2008) Improvement of the dermal epidermal junction in human reconstructed skin by a new c-xylopyranoside derivative. Eur J Dermatol 18: 297–302. 18474459 10.1684/ejd.2008.0392

[29] Muto J, Naidu NN, Yamasaki K, Pineau N, Breton L, et al (2011) Exogenous addition of a C-xylopyranoside derivative stimulates keratinocyte dermatan sulfate synthesis and promotes migration. PLoS One 6: e25480. 21998662 10.1371/journal.pone.0025480 PMC3187761

[30] Wasteson A (1971) A method for the determination of the molecular weight and molecular-weight distribution of chondroitin sulphate. J Chromatogr 59: 87–97. 5110295 10.1016/s0021-9673(01)80009-1

[31] Lyon M, Deakin JA, Gallagher JT (1994) Liver heparan sulfate structure. A novel molecular design. J Biol Chem 269: 11208–11215. 8157650

[32] Passi A, Albertini R, Campagnari F, De Luca G (1997) Modifications of proteoglycans secreted into the growth medium by young and senescent human skin fibroblasts. FEBS Lett 402: 286–290. 9037211 10.1016/s0014-5793(97)00008-2

[33] Shin JE, Oh JH, Kim YK, Jung JY, Chung JH (2011) Transcriptional regulation of proteoglycans and glycosaminoglycan chain-synthesizing glycosyltransferases by UV irradiation in cultured human dermal fibroblasts. J Korean Med Sci 26: 417–424. 21394312 10.3346/jkms.2011.26.3.417 PMC3051091

[34] Vuillermoz B, Wegrowski Y, Contet-Audonneau JL, Danoux L, Pauly G, et al (2005) Influence of aging on glycosaminoglycans and small leucine-rich proteoglycans production by skin fibroblasts. Mol Cell Biochem 277: 63–72. 16132716 10.1007/s11010-005-5073-x

[35] Su X, Paris M, Gi YJ, Tsai KY, Cho MS, et al (2009) TAp63 prevents premature aging by promoting adult stem cell maintenance. Cell Stem Cell 5: 64–75. 19570515 10.1016/j.stem.2009.04.003 PMC3418222

[36] Takamiya M, Biwasaka H, Saigusa K, Nakayashiki N, Aoki Y (2009) Wound age estimation by simultaneous detection of 9 cytokines in human dermal wounds with a multiplex bead-based immunoassay: an estimative method using outsourced examinations. Leg Med (Tokyo) 11: 186–190. 19419898 10.1016/j.legalmed.2009.03.010

[37] Birchmeier C, Birchmeier W, Gherardi E, Vande Woude GF (2003) Met, metastasis, motility and more. Nat Rev Mol Cell Biol 4: 915–925. 14685170 10.1038/nrm1261

[38] Mildner M, Mlitz V, Gruber F, Wojta J, Tschachler E (2007) Hepatocyte growth factor establishes autocrine and paracrine feedback loops for the protection of skin cells after UV irradiation. J Invest Dermatol 127: 2637–2644. 17597814 10.1038/sj.jid.5700938

[39] Ashikari-Hada S, Habuchi H, Kariya Y, Itoh N, Reddi AH, et al (2004) Characterization of growth factor-binding structures in heparin/heparan sulfate using an octasaccharide library. J Biol Chem 279: 12346–12354. 14707131 10.1074/jbc.M313523200

[40] Catlow KR, Deakin JA, Wei Z, Delehedde M, Fernig DG, et al (2008) Interactions of hepatocyte growth factor/scatter factor with various glycosaminoglycans reveal an important interplay between the presence of iduronate and sulfate density. J Biol Chem 283: 5235–5248. 18156180 10.1074/jbc.M706589200

[41] Lyon M, Deakin JA, Mizuno K, Nakamura T, Gallagher JT (1994) Interaction of hepatocyte growth factor with heparan sulfate. Elucidation of the major heparan sulfate structural determinants. J Biol Chem 269: 11216–11223. 8157651

[42] Deakin JA, Blaum BS, Gallagher JT, Uhrin D, Lyon M (2009) The binding properties of minimal oligosaccharides reveal a common heparan sulfate/dermatan sulfate-binding site in hepatocyte growth factor/scatter factor that can accommodate a wide variety of sulfation patterns. J Biol Chem 284: 6311–6321. 19114710 10.1074/jbc.M807671200

[43] Bernerd F, Asselineau D (1997) Successive alteration and recovery of epidermal differentiation and morphogenesis after specific UVB-damages in skin reconstructed in vitro. Dev Biol 183: 123–138. 9126289 10.1006/dbio.1996.8465

[44] Black AF, Bouez C, Perrier E, Schlotmann K, Chapuis F, et al (2005) Optimization and characterization of an engineered human skin equivalent. Tissue Eng 11: 723–733. 15998214 10.1089/ten.2005.11.723

[45] Hiscock DR, Canfield A, Gallagher JT (1995) Molecular structure of heparan sulphate synthesised by bovine aortic endothelial cells. Biochim Biophys Acta 1244: 104–112. 7766644 10.1016/0304-4165(94)00206-d

[46] Vives RR, Sadir R, Imberty A, Rencurosi A, Lortat-Jacob H (2002) A Kinetics and Modeling Study of RANTES(9–68) Binding to Heparin Reveals a Mechanism of Cooperative Oligomerization. Biochemistry 41: 14779–14789. 12475226 10.1021/bi026459i

